# Post-translational Modification-Based Regulation of HIV Replication

**DOI:** 10.3389/fmicb.2018.02131

**Published:** 2018-09-11

**Authors:** Lin Chen, Oliver T. Keppler, Christian Schölz

**Affiliations:** Max von Pettenkofer-Institute and Gene Center, Virology, National Reference Center for Retroviruses, Faculty of Medicine, Ludwig-Maximilians-University Munich, Munich, Germany

**Keywords:** HIV, post-translational modification, PTM, viral replication, HIV life cycle

## Abstract

Human immunodeficiency virus (HIV) relies heavily on the host cellular machinery for production of viral progeny. To exploit cellular proteins for replication and to overcome host factors with antiviral activity, HIV has evolved a set of regulatory and accessory proteins to shape an optimized environment for its replication and to facilitate evasion from the immune system. Several cellular pathways are hijacked by the virus to modulate critical steps during the viral life cycle. Thereby, post-translational modifications (PTMs) of viral and cellular proteins gain increasingly attention as modifying enzymes regulate virtually every step of the viral replication cycle. This review summarizes the current knowledge of HIV-host interactions influenced by PTMs with a special focus on acetylation, ubiquitination, and phosphorylation of proteins linked to cellular signaling and viral replication. Insights into these interactions are surmised to aid development of new intervention strategies.

## Introduction

Multiple advances in HIV pharmacotherapy have been made allowing control of viremia, but not eradication of latent viral reservoirs. To cope this difficulty, new promising efforts such as gene therapy (Peterson and Kiem, 2017), utilization of broadly neutralizing antibodies (immunotherapy) (Nishimura and Martin, 2017) or the development of first potential vaccines (Barouch et al., 2018) have been applied. Nevertheless, it remains a high priority to enhance our understanding of HIV pathogenesis, latency and persistence in anatomical, immunological and pharmacological sanctuaries to develop novel drugs/strategies for the treatment and eradication of HIV.

In the last few years, substantial progress has been made toward understanding of biomolecular mechanisms underlying the interaction of the virus with the host cell. New methods have allowed deeper insights into the “rewiring” of cellular pathways by the invading virus. In particular the identification of PTMs helped to understand how HIV adjusts cell cycle, transcription, translation or selective degradation of antiviral proteins. The importance of PTMs in HIV infection is highlighted by several studies that successfully used modifying enzyme inhibitors for reduction of viral load or reactivation of latent virus. Examples are the use of *proto-oncogene tyrosine-protein kinase Src* (SRC)- or lysine-deacetylase inhibitors (Shirakawa et al., 2013; McCarthy et al., 2014). However, despite the significance of PTMs for HIV, a comprehensive overview of PTM-based HIV-host interactions is missing so far.

**FIGURE 1:**
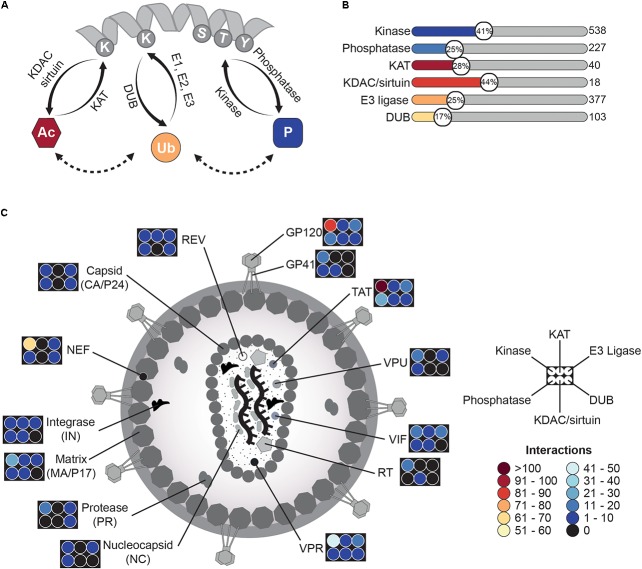
HIV-related PTMome – an overview. **(A)** Schematic illustration of protein modifications discussed in this review. Proteins can be modified by different enzymes, which either introduce a functional group (“writers”) or remove it (“erasers”). Thereby, lysine acetylation is achieved by lysine-acetyltransferases (KATs) and the backward reaction is fulfilled by lysine deacetylases (KDACs, also known as histone deacetylases (HDACs)) or sirtuins. Lysines can be also modified by ubiquitin in presence of E1 ubiquitin-activating-, E2 ubiquitin-conjugating-enzymes, and E3 ubiquitin ligases. Reversal of ubiquitin linkages is achieved by deubiquinating enzymes (DUBs). Another frequent modification occurs at serine-/threonine- and/or tyrosine-residues, where phosphate groups are transferred to by kinases. Removal of phosphorylation is carried out by phosphatases. All three modifications can influence each other and might occur at the same protein. **(B)** HIV interacts with several modifying enzymes. Based on the NCBI HIV interaction database, several interactions of viral proteins with modifying enzymes have been described. Shown here are six groups of writers and erasers, which interact in one way or another with HIV during the viral lifecycle. The graph provides the percentage (number in circle) of interacting enzymes of a respective class in comparison to the amount of enzymes of the same class found in the human proteome. Number of all enzymes of a class is given on the right site and is based on the HNGC database, the National Heart Lung and Blood Institute ESBL human E3 ubiquitin ligases databank, The Ubiquitin and Ubiquitin-like Conjugation Database (UUCD), and databases for protein kinases^2^ and phosphatases (DEPOD)^3^. Notably, for acetylation not only lysine-acetyltransferases but also N-terminal acetyltransferases were included. **(C)** Schematic overview of viral proteins interacting with modifying enzymes. The graph depicts the number of interactions of different HIV proteins with kinases/phosphatases, KATs/KDACs, and E3 ligases/DUBs. Number of interactions is provided in form of a heat map.

In this review, we attempt to summarize current knowledge of modification-controlled processes during HIV replication, emphasizing several key interactions among viral and cellular components. Given the broad scope and the overwhelming amount of available data, we chose to focus on PTM-related interactions, which might be of greater interest. Thus, we spotlight on protein acetylation, ubiquitination, and phosphorylation events (**Figure 1A**) and their respective role within the different steps of the viral life cycle. As main foundation and data source, we made use of the NCBI HIV Human Interaction Database (HHID) (Ako-Adjei et al., 2015), which harbors more than 8000 interactions and more than 6800 corresponding publications. Notably, some of the described interactions are derived from systems-wide mass spectrometry and next generation sequencing approaches that still require experimental confirmation. Nonetheless, in combination with orthogonal methods and experimental/clinical observations, they provide an exceptional overview of the intricate relationship between HIV and the human host.

## Ptms and Hiv Life Cycle – a Close Interrelationship

To gain a first overview of the interactions between HIV and the host-related PTM machinery, we searched HHID (Ako-Adjei et al., 2015) and cross-correlated data with other data repositories to identify interactions of HIV proteins with post-translational modifying enzymes.

For identification of human acetyltransferases and deace-tylases, we utilized the HUGO Gene Nomenclature Committee (HGNC) database ^1^, resulting in eleven lysine- and N-terminal-acetyltransferases out of 40 listed (28%) and eight lysine deacetylases (KDACs and sirtuins) out of 18 (44%). In case of E3 ubiquitin ligases, the NHLBI ESBL human E3 ubiquitin ligases database (Medvar et al., 2016) served as data source and correlation with the HHID showed interaction with 95 E3 ligases out of 377 (25%). The Ubiquitin and Ubiquitin-like Conjugation Database (UUCD) (Gao T. et al., 2013) revealed overall 18 deubiquitinating enzymes (DUBs) out of 103 (17%), which have been identified so far for HIV-related interactions. The most comprehensive data were achieved for protein kinases ^2^ (Manning et al., 2002) and phosphatases ^3^ (Duan et al., 2015), as phosphorylation has been more extensively studied. Here, 219 kinases out of 538 (41%) and 57 phosphatases out of 227 (25%), respectively, turned out to be influenced during the HIV life cycle (**Figure 1B**). Further analysis of the various datasets and the HIV interaction repository indicated several viral hubs, which extensively modulate the human host PTMome. To mention some of them, HIV-1 envelope glycoprotein 120 (GP120) has been found to interact or functionally alter 118 enzymes of the aforementioned classes, for the HIV-1 *trans-activator of transcription* (TAT) 155 enzymes and for the *viral protein R* (VPR) 73 enzymes (**Figure 1C**) have been described. Overall, the HHID lists so far 138 acetyl-, 1899 phospho-, and 359 ubiquitin-related HIV-host interactions.

In the following chapters, we will dissect some important interactions considering the different steps of the viral life cycle – from viral entry to budding of newly generated virions.

### Virus Binding

**FIGURE 2:**
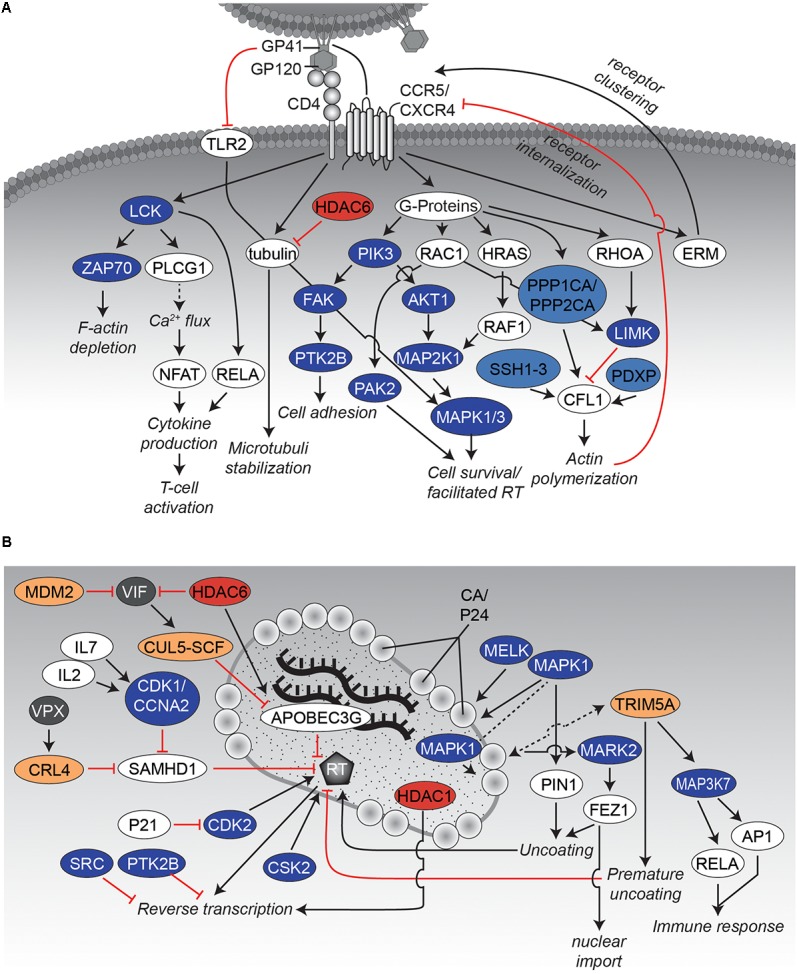
Interaction networks of HIV and host proteins during early steps of infection. **(A)** Binding of HIV to the host cell. Binding of the viral envelope GP160 complex, consisting of GP120 and GP41, to CD4 as well as the co-receptors CXCR4 or CCR5 results in activation of several protein kinases. Thereby, the cytoskeleton becomes rearranged, which favors entry and subsequent nuclear import of the virus. The network displays an overview of relevant interactions. Black arrows indicate activating effects and red “inhibitory”-arrows indicate inhibitory effects. Kinases are depicted in dark blue, phosphatases in light blue, KATs in dark red, KDACs in light red, E3 ligases in dark yellow, and DUBs in light yellow. Other host proteins are colored white, whereas viral proteins are presented in gray. **(B)** Uncoating and reverse transcription. The graph illustrates several protein-protein interactions that have been described during uncoating and reverse transcription. Please note that VPX is only expressed in HIV-2 and SIV but not in HIV-1. CRL4 complex consists of *cullin4A* (CUL4A*), DNA damage binding protein 1* (DDB1), *RING H2 finger protein* (RBX1) and the *DDB1 and* *CUL4-associated Factor 1* (DCAF1). Figure legend as in **A**. More detailed information can be found in the text.

The initial step of HIV infection depends on adhesion of the virion to the target host cell and subsequent fusion of viral and host cell membranes. Whereas initial binding of virus particles to the host cell is a relatively unspecific process and relies mainly on weak protein-protein interactions, the second step consists of a highly controlled sequence of events that are crucial for virion entry (**Figure 2A**). Thereby, the HIV *envelope* (ENV) spike complex, consisting of glycoprotein 41 (GP41)/GP120 trimers, binds to the *T-cell surface glycoprotein CD4* (CD4) receptor on the host cell. Subsequently, conformational changes of ENV/GP120 subunits allow binding to the coreceptors *C-C chemokine receptor type 5* (CCR5) or *C-X-C chemokine receptor type 4* (CXCR4) (Maddon et al., 1986; McDougal et al., 1986). During this process, HIV already modulates multiple intracellular signaling cascades in a PTM-specific manner by mimicking chemokine signaling through binding to their cognate receptors (Wu and Yoder, 2009). Most of these modifications result in the highly dynamic (re-)organization of the cytoskeleton (**Figure 2A**). One can assume that these rearrangements are necessary to allow not only fusion of the viral particle with the cell, but also prepare for later intracellular transport. As an example, G-protein related downstream signaling pathways are activated upon virus binding affecting e.g., the cytoskeleton structure, cytokine production and T-cell activation (Abbas and Herbein, 2014). Stimulation of lymphocytes with GP120 activates *mitogen-activated protein kinase 1* and *3* (MAPK1/3) via *dual specificity mitogen-activated protein kinase kinase 1* (MAP2K1) as well as *protein-tyrosine kinase 2-beta* (PTK2B) by interaction with glycosphingolipids (Viard et al., 2003). Activation of the PTK2B signaling pathways finally leads to rearrangements of the cytoskeleton (Abbas and Herbein, 2014). Concurrently, stimulation of MAPK1/3 triggers e.g., survival of the T-cell. Notably, the MAPK-pathway is not activated in quiescent T-cells or non-proliferating CD4 T-cells, and thus its induction is dependent on the activation state of the target T-cell (Kinet et al., 2002). Additionally, ENV/GP120 induces phosphorylation of the SRC-family member *tyrosine-protein kinase Lck* (LCK) (Juszczak et al., 1991; Deng et al., 2016), which stimulates nuclear translocation of *transcription factor p65* (RELA; also known as NF-κB) (Flory et al., 1998), being required for later HIV-1 long terminal repeat (LTR)-dependent transcription. LCK activation induces phosphorylation of *tyrosine kinase ZAP-70* (ZAP70) as well as of numerous cellular substrates leading to the depletion of cortical F-actin underneath the virological synapse to ease transmission of the viral core to the nucleus after viral entry (Vasiliver-Shamis et al., 2009; Len et al., 2017). Moreover, LCK activates *1-phosphatidylinositol 4,5-bisphosphate phosphodiesterase gamma-1* (PLCG1), resulting in upregulated Ca^2+^ flux and activation of the transcription factor *nuclear factor of activated T-cells* (NFAT) (Abbas and Herbein, 2014). This way not only several cytokines like *interleukin 2* (IL2) are expressed, which trigger activation of the T-cell, but also viral replication in sub-optimally activated cells might be promoted (Cicala et al., 2006). Within the context of actin rearrangements, binding of GP120 to CXCR4 also triggers *transforming protein* *RhoA* (RHOA) and *Ras-related C3 botulinum toxin* *substrate 1* (RAC)-dependent pathways causing phosphorylation of actin severing factor *cofilin-1* (CFL1) by *LIM domain kinase* (LIMK), and thus the transient inactivation of CFL1 (Jiménez-Baranda et al., 2007). Consequently, actin remodeling is reduced which might contribute to a more efficient receptor clustering (Stolp and Fackler, 2011). In the subsequent steps during virus entry, a rapid switch from an inactivated to an activated state of CFL1 by dephosphorylation is established, which may involve several phosphatases, like *protein phosphatase slingshot homolog 1-3* (SSH1-3), *pyridoxal phosphate phosphatase* (PDXP), *serine/threonine-protein phosphatases PP1-alpha* (PPP1CA) and *PP2-alpha* (PPP2CA) (Wu et al., 2008). Activated CFL1 induces actin polymerization, which might contribute to overcome static actin restriction and to block CXCR4 internalization (Yoder et al., 2008; Spear et al., 2012).

Previous studies identified other actin-related factors, such as the cytoskeletal crosslinking factor proteins *ezrin* (EZR), *radixin* (RDX) and *moesin* (MSN) (also named together ERM), that show increased phosphorylation upon interaction of HIV ENV/GP120 with CXCR4 or CCR5. However, the distinct roles of ERM proteins on the early phase of HIV-1 replication have been controversial, as positive (Kubo et al., 2008; Barrero-Villar et al., 2009) and negative (Naghavi et al., 2007; Capalbo et al., 2011) effects on HIV have been described.

Besides several phosphorylation events, lysine (de-)acetylation has been also identified as a regulating PTM for the reorganization of the cytoskeleton: Binding of ENV/GP120 to CD4 induces the formation of acetylated α-tubulin which is involved in stabilization of microtubules and thus fosters HIV infection (Piperno et al., 1987; Valenzuela-Fernández et al., 2005; Sabo et al., 2013). Conversely, cytosolic *histone deacetylase 6* (HDAC6) has been found to counteract tubulin acetylation and thereby suppressing infection by HIV (Valenzuela-Fernández et al., 2005). An overview of the described events is given in **Figure 2A**.

### Fusion, Uncoating and Reverse Transcription

Conformational changes finally cause dissociation of GP120 from GP41 and the transition of GP41 into its fusogenic state. It is believed that a so far not completely understood combination of interactions finally leads to the formation of the GP41 six-helix bundle and along with that the fusion of the virion with the host cell membrane. Interactions are surmised to include GP41, virus- and target cell-proteins, accompanied cytoskeletal rearrangements (as discussed in more detail in the previous chapter), and lipids.

As soon as the virus has entered the cell, its conical capsid core is released into the cytoplasm. At this time point, reverse transcription of the viral RNA into double-stranded cDNA is initiated (**Figure 2B**). Concurrently, dissociation of the protective, conical capsid surrounding the HIV1 genome occurs, a process known as uncoating. However, the detailed spatial and temporal steps of uncoating and reverse transcription remain unsolved. Three potential models for the uncoating process of HIV-1 have been advanced so far [for further information of the uncoating process, we would like to refer to a review from (Campbell and Hope, 2015)]. In brief, the “immediate uncoating” model describes the rapid disassembly of the viral core shortly after membrane fusion (Miller et al., 1997; Fassati and Goff, 2001). Thereby, the reverse transcription complex (RTC), consisting of viral *reverse transcriptase* (RT), *integrase* (IN), viral RNA and newly synthesized viral DNA, exists freely in the cytoplasm loosely surrounded by viral capsid proteins (CA/P24) and is transported toward the nucleus. Several findings suggest that CA/P24 as well as the capsid core itself provide critical functions for reverse transcription of viral RNA and its nuclear transport promoting two other models of uncoating. In the first model the core remains associated with the RTC (Perez-Caballero et al., 2005; Hulme et al., 2011; Lukic et al., 2014), mediating the interaction with host factors and the nuclear import (“cytoplasmic uncoating”). In the second model the core stays intact until arrival at a nuclear pore complex (“NPC uncoating”), preventing detection of viral double-stranded DNA by cytosolic DNA sensors like the *cyclic GMP-AMP synthase* (CGAS) (Gao D. et al., 2013; Hilditch and Towers, 2014). The later model is further supported by the recent discovery of dynamic capsid pores for nucleotide import (Jacques et al., 2016). Independent of the respective model, it is believed that the disassembly of the viral core underlies a controlled reaction. It has been demonstrated that the RTC/pre-integration complex (PIC) as well as parts of the capsid are transported via the microtubule network toward the nucleus (McDonald et al., 2002; Fernandez et al., 2015). Experimental evidence has been reported that CA/P24 as well as the capsid core structure harbor critical functions for reverse transcription, uncoating, and nuclear localization.

Several of these functions are regulated via PTMs (**Figure 2B**). As an example, CA/P24 becomes phosphorylated at three serine residues (S109, S149, S178) affecting the reverse transcription process (Cartier et al., 1999). Interestingly, the function of virions carrying mutated serine residues S149 and S178 could be rescued by pseudotyping the virus with *vesicular stomatitis virus glycoprotein* (VSV-G) to bypass HIV envelope fusion (Brun et al., 2008). Takeuchi and colleagues recently identified *maternal embryonic zipper kinase* (MELK) to be responsible for at least phosphorylation of serine S149 and this modification appears to be critical for optimal uncoating and viral cDNA synthesis (Takeuchi et al., 2017). Other studies identified MAPK1 to mediate phosphorylation of serine S16 of CA/P24 and the regulation of *peptidyl-prolyl cis-trans isomerase NIMA-interacting-1* (PIN1)-dependent uncoating (Misumi et al., 2010; Dochi et al., 2014). The spatiotemporal interaction of MAPK1 with CA/P24 is under debate, as MAPK1 has also been identified as virion associated protein kinase. Low level activity of MAPK1 might already exist within the virion and is expected to be enhanced by stimuli accompanied with binding and fusion of the virion to its target cell (Bukrinskaya et al., 1996; Cartier et al., 1999).

Parallel to the uncoating of the viral core, the reverse transcription of the viral genome takes place (**Figure 2B**). This process is regulated by several protein-protein interactions as well as PTMs critical for efficient viral cDNA generation. In addition, PTMs are important to counteract transcription by activation/modulation of several host restriction factors. During the binding of the virion to the host cell, interactions with the CD4 receptor as well as the co-receptors CXCR4 or CCR5 induce the Gαi-dependent MAPK pathway via phosphorylation, which facilitates reverse transcription (Mettling et al., 2008). Moreover, several other modifications have been demonstrated to be required after fusion for modulation of RT activity. In this context, host *cyclin-dependent kinases* (CDKs) have been identified as influencers of RT activity. As an example, CDK2 directly phosphorylates RT on tyrosine residue T216, thereby increasing its efficacy and stability (Leng et al., 2014). Interestingly, phosphorylation of T216 was abolished by cell-intrinsic CDK2 inhibitor *cyclin-dependent kinase inhibitor 1A* (CDKN1A, also known as P21), which has been shown to be upregulated in “elite-controller” patients (Chen et al., 2011; Sáez-Cirión et al., 2011; Leng et al., 2014).

Moreover, *casein kinase II* (CSK2) has been shown to be responsible for RT phosphorylation, as investigated in HIV-1 infected MOLT-4 cells (Ohtsuki et al., 1998). Phosphorylation of purified RT *in vitro* by several kinases including CSK2 was also shown by the group of S. Wilson (Idriss et al., 1999). The significance of RT phosphorylation by CSK2 was further corroborated by another study, demonstrating that the different molecular functions (RNA-, DNA-dependent polymerase, ribonuclease H) of RT were stimulated *in vitro* via CSK2-dependent phosphorylation (Harada et al., 1999).

### Reverse Transcription and Antiviral Restriction

HIV evolved direct and indirect ways to counteract host cellular restriction factors that block viral RT activity (**Figure 2B**). For instance, HIV-1-induced lymphopenia is associated with increased levels of *interleukin 7* (IL7) (Napolitano et al., 2001). IL7 is able to induce phosphorylation of *deoxynucleoside triphosphate triphosphohydrolase SAMHD1* (SAMHD1) via the *cyclin A2* (CCNA2)/ c*yclin dependent kinase 1* (CDK1) complex (Cribier et al., 2013; Coiras et al., 2016), resulting in the loss of SAMHD1’s ability to restrict reverse transcription via reduction of the intracellular pool of dNTPs (Lahouassa et al., 2013). Notably, phosphorylation and dephosphorylation of SAMHD1 as well as its overall expression levels are modulated also independently of IL7 throughout the cell cycle. SAMHD1 is highly expressed during quiescence (G 0 phase) but minimally expressed and present in an phosphorylated, inactive state during replication (S phase) (Sze et al., 2013).

Besides the indirect control of SAMHD1 via IL7, several simian immunodeficiency virus (SIV) groups and their closely related HIV-2 counterparts carry the accessory *viral protein X* (VPX) (which is missing in HIV-1) for directly targeting SAMHD1 (Laguette et al., 2011; Hofmann et al., 2012). Thereby, VPX interacts with the *cullin4A* (CUL4A)/ *DNA damage binding protein 1* (DDB1) *and CUL4-associated Factor 1* (DCAF1) complex (CRL4 complex), leading to ubiquitination and subsequent proteasomal degradation of SAMHD1. VPX induced degradation of SAMHD1 might not only affect the cytosolic dNTP pool, but is suggested also to influence the concentration of nuclear dNTP levels required for gap repair in HIV integration. Moreover, it prevents SAMHD1’s ability to bind long RNAs/single-stranded DNA, which might as well restrict reverse transcription (Seamon et al., 2016).

Another host restriction factor counteracted by HIV within this process is *DNA dC -> dU-editing enzyme APOBEC-3G* (APOBEC3G). APOBEC3G exerts a multifaceted inhibition scheme against reverse transcription as it deaminates cytosine residues to uracil in viral minus strand DNA, resulting in hypermutations of the provirus (Harris et al., 2003; Bishop et al., 2004). This activity of APOBEC3G is so effective that even a single incorporated APOBEC3G-unit is likely to cause extensive and inactivating levels of HIV hypermutations (Zhang et al., 2003; Armitage et al., 2012). Concurrently, APOBEC3G significantly inhibits RT-catalyzed DNA elongation (Iwatani et al., 2007). Here, the HIV-accessory protein *virion infectivity factor* (VIF) induces polyubiquitination and subsequental proteasomal degradation of APOBEC3G via recruitment of the *cullin-5* (CUL5)- *SKP, Cullin, F-box containing* (SCF) complex (Yu, 2003; Goncalves and Santa-Marta, 2004). Simultaneously, VIF is in turn degraded by the host: The *E3 ubiquitin-protein ligase Mdm2* (MDM2) was reported to post-translationally modify VIF, thus targeting it for proteasomal destruction (Izumi et al., 2009).

Similarly, HDAC6 can antagonize VIF activity by promoting autophagic clearance of the viral accessory protein via its deacetylase activity. Additionally, there is evidence that HDAC6 is able to reduce the amount of VIF incorporated into nascent virions and thus affects infectivity of HIV particles. Moreover, HDAC6 directly binds to and stabilizes APOBEC3G, resulting in impaired reverse transcription (Valera et al., 2015). In contrast to the mentioned restrictive effect of HDAC6 on reverse transcription, another deacetylase has been identified to be required for reverse transcription. Yeast-two-hybrid screening identified *histone deacetylase complex subunit SAP18* (SAP18) and components of the *paired amphipathic helix protein Sin3a* (SIN3A)-*histone deacetylase 1* (HDAC1) complex to be interacting with IN (Sorin et al., 2009). Further experiments revealed incorporation of HDAC1 into newly formed HIV-1 virions and showed that enzymatically inactive mutants of HDAC1 result in a block of early reverse transcription.

Another example of antiviral defense of the host cell is the activation of protein tyrosine kinases shortly after binding of the virus. Two of these kinases are SRC and PTK2B, which are known to interact with each other. Experiments with kinase inhibitors and RNA interference targeting SRC in human T-cells revealed increased HIV-1 reverse transcription, genomic integration, and subsequent gene transcription. Thus, activation of SRC might be part of a cellular response system to prevent or at least slow down infection (McCarthy et al., 2014).

In summary, all in this chapter and the one before mentioned examples (please see also **Figure 2B**) demonstrate the impact of PTMs on the early steps of viral infection, where both, virus and host make use of specific modifications to modulate protein machineries and signaling pathways to either support or counteract reverse transcription.

### Nuclear Import and Integration

Within the course of uncoating and reverse transcription of the viral single-stranded RNA, newly synthesized DNA associates/remains associated with viral and cellular proteins forming the preintegration complex (PIC). The PIC consists of the newly transcribed viral cDNA, The viral proteins IN, RT, VPR, and HIV matrix protein (MA/P17) as well as an incompletely characterized group of host factors (Fassati and Goff, 2001; Lin and Engelman, 2003; Llano et al., 2004b; Iordanskiy et al., 2006; Raghavendra et al., 2010; Matreyek and Engelman, 2013). Utilizing the cellular nuclear import machinery, HIV cDNA as part of the PIC is actively delivered into the nucleus for subsequent integration and productive infection. As a consequence, HIV can successfully replicate even in non-dividing, cell-cycle arrested cells (Lewis et al., 1992) and in terminally differentiated cells like macrophages or dendritic cells (Gabuzda et al., 1986; Patterson and Knight, 1987; Weinberg et al., 1991; Pope et al., 1994; Reece et al., 1998).

**FIGURE 3:**
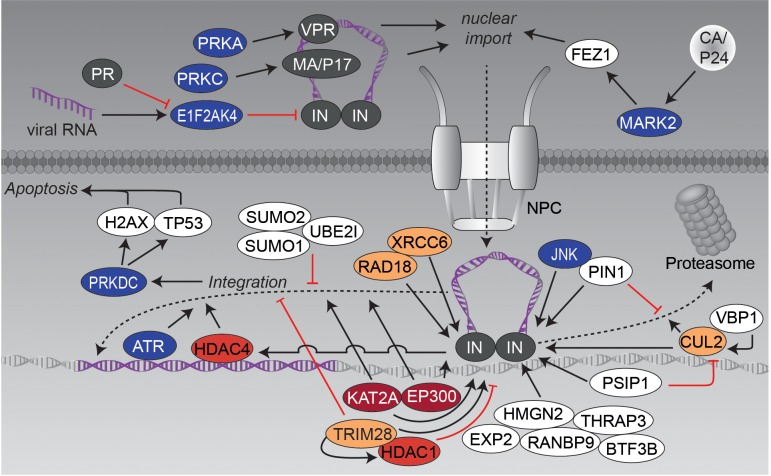
Nuclear import and genomic integration. The graphic illustrates the influence of post-translational modifying enzymes on the nuclear transport of the PIC and the integration of the viral cDNA into the host genome. Figure legend as in **Figure 2A**. More detailed information can be found in the text.

The PIC’s route into the nucleus is accompanied by several tightly regulated PTM-based interactions, which will be discussed within this chapter based on different examples (see also **Figure 3**). The first example is the kinesin-1 adaptor *fasciculation and elongation protein zeta-1* (FEZ1), which is exploited by the HIV-1 capsid for its movement into the nucleus (Malikov et al., 2015). It has been shown that FEZ1 and kinesin-1 heavy, but not light chains affect not only HIV-1 transport, but also the uncoating process. Thereby, the capsid binds *serine/threonine-protein kinase MARK2* (MARK2, also known as *microtubule affinity regulating kinase 2*) to stimulate FEZ1 phosphorylation (Malikov and Naghavi, 2017).

Another, highly modified and extensively examined protein in this context is IN, which is involved in different steps of HIV replication such as reverse transcription, nuclear import, genomic integration and also later on in virion packaging. Recently, *eukaryotic translation initiation factor-2-alpha kinase 4* (EIF2AK4, also known as GCN2) has been identified as a general restriction factor of lentiviral DNA integration (Jaspart et al., 2017). Infection with HIV-1 triggers acute decrease in protein translation by phosphorylation and thereby activation of EIF2AK4. Moreover, EIF2AK4 directly interacts with IN causing phosphorylation of serine S255 in its C-terminal domain (CTD), which decreases efficiency of viral DNA integration. Antiviral activity of EIF2AK4 is counteracted by HIV-1 and HIV-2 proteases (PR), which cause proteolytic cleavage of EIF2AK4 and thus abrogate activation of EIF2AK4 by viral RNA (del Pino et al., 2012; Cosnefroy et al., 2013; Jaspart et al., 2017). In activated T-cells, IN becomes phosphorylated on serine S57 by cellular *c-Jun N-terminal kinase* (JNK). Subsequently, phosphorylated IN is conformationally modified by *peptidylprolyl cis/trans isomerase, NIMA-interacting 1* (PIN1), which stabilizes IN (blockade of ubiquitination and associated proteasomal degradation) and seems to be required for efficient genomic integration. Lack of this modification restricts viral infection in non-activated/resting CD4 T-cells (Manganaro et al., 2010).

Using the translation inhibitor cycloheximide, as well as proteasome inhibitor MG132, it was demonstrated that IN, overexpressed as single protein in HEK293T cells, is relatively unstable and is poly-ubiquitinated for proteasomal degradation (Mulder and Muesing, 2000). Another study identified the *prefoldin subunit 3* (VBP1, also known as PFDN3 or *von Hippel-Lindau-binding protein 1*) as interacting protein, that bridges IN and *cullin-2* (CUL2)-related *von Hippel-Lindau disease tumor suppressor* (VHL) ubiquitin E3 ligase for degradation (Mousnier et al., 2007). Interestingly, degradation seems to be important for later viral replication as a 60% reduction of released infectious particles was observed mutating degradation-relevant lysines K211, K215 and K219 (Mousnier et al., 2007). Degradation of IN is circumvented by different host cell proteins: e.g., *PC4 and SFRS1-interacting prote*in (PSIP1, also known as LEDGF) was identified as interacting with IN and blocking VHL-based degradation (Cherepanov et al., 2003; Llano et al., 2004a). Other approaches identified human *E3-ubiquitin protein ligase RAD18* (RAD18) as well as *X-ray repair cross-complementing protein 6* (XRCC6), both known for their role in DNA repair, as IN stabilizing factors (Mulder et al., 2002; Zheng et al., 2011). However, the exact roles of RAD18 and XRCC6 in HIV replication remain elusive.

Besides ubiquitination, IN has been shown to be modified by *small ubiquitin-like modifier* (SUMO), a PTM that is involved in various cellular processes including the transport between cytosol and nucleus or the transcriptional regulation. Several conserved lysine residues of IN were analyzed to have an impact on HIV-1 replication after reverse transcription, but before genomic integration (Zamborlini et al., 2011). The researchers hypothesized that these modifications might regulate interactions with different co-factors, harboring *sumo-interacting motif* (SIM) domains, like *tripartite motif-containing protein 5 alpha* (TRIM5A). This notion is also supported by findings that IN-interacting proteins like PSIP1 or RAD18 are involved in SUMO-related protein-protein complexes (Bueno et al., 2010; Despras et al., 2016). Furthermore, overexpression of SUMO1/SUMO2 and *SUMO-conjugating enzyme UBC9* (UBE2I) perturbed intracellular localization of IN and influenced integration of HIV-1, while reverse transcription and nuclear import of the PIC remained unaffected (Li et al., 2012).

Immuno-precipitations and fluorescence resonance energy transfer (FRET) experiments revealed another modification of IN – namely lysine-acetylation by *histone acetyltransferase p300* (EP300) at lysine residues K264, K266, and K273 (Cereseto et al., 2005). Acetylation increased the affinity of IN to DNA and promoted its ability for DNA strand transfer. The importance of lysine-acetylation was further demonstrated by acetylation-incompetent mutants, which caused a suppression of viral integration capacity. The same group as well identified the *histone acetyltransferase KAT2A* (KAT2A, also known as GNC5) as interactor and modifier of IN (Terreni et al., 2010). KAT2A-related acetylation of IN enhanced its 3^′^-end processing and strand transfer activity. Knockdown of KAT2A markedly reduced HIV-1 infectivity. Interestingly, KAT2A targets the same lysines as EP300 with exception of K258, which is acetylated by KAT2A only. Notably, importance of IN acetylation was questioned by lysine to arginine mutations of the three lysines, controlled by EP300 and KAT2A, since the mutated viruses were still replication-competent (Topper et al., 2007).

A yeast-two-hybrid screen identified, amongst others, PSIP1, *transcription intermediary factor 1-beta* (TRIM28, also known as TIF1B), *transcription factor BTF3* (BTF3B), *thyroid hormone receptor-associated protein 3* (THRAP3), *non-histone chromosomal protein HMG-17* (HMGN2), *ran-binding protein 9* (RANBP9), and *exportin-2* (EXP2) as binding partners of acetylated IN (Allouch and Cereseto, 2011). Further experiments confirmed direct binding of TRIM28 to IN and demonstrated recruitment of HDAC1, which resulted in deacetylation of IN. TRIM28 seems to reduce IN-dependent genomic integration and thus restricts HIV-1 infection (Allouch et al., 2011). Acetylation has been implicated in the process of post-integration repair. The group of R. Daniel was able to show that, on the one hand, *histone deacetylase 4* (HDAC4) associates with viral DNA in an IN-dependent manner and, on the other hand, seems to be required for efficient transduction by HIV-1-based vectors in cells that are deficient in other DNA repair proteins (Smith and Daniel, 2006; Smith et al., 2010). Further research is required to clarify the impact of lysine acetylation in IN activity and in general on HIV infection.

Besides IN, other viral protein functions are PTM-modulated during the integration process of HIV-1. For instance, the MA/P17 is phosphorylated on tyrosine Y132 by a not further characterized virion-associated cellular protein kinase, which targets MA/P17 to the nucleus during transversion of the PIC. Interestingly, viruses carrying MA/P17 tyrosine mutants grow normally in dividing cells, but are blocked for nuclear import in terminally differentiated cells like macrophages (Gallay et al., 1995). MA/P17 interacts with *protein kinase C* (PRKC) resulting in phosphorylation of serine residue S111 (Burnette et al., 1993). Phosphoaminoacid and phosphopeptide analysis also revealed phosphorylation of other serine-residues of MA/P17 – especially in nuclear fractions of infected cells (Bukrinskaya et al., 1996). However, the time and exact role of MA/P17 phosphorylation in the context of nuclear import and integration is still controversial as two reports indicated that its phosphorylation predominately occurs before virion entry (Gallay et al., 1995; Bukrinskaya et al., 1996).

An intriguing link between integration and pathogenesis was suggested by a study showing that infection by wild-type HIV-1, but not an integrase-deficient mutant, induced the death of activated primary CD4 T-lymphocytes. The mechanism of killing involves activation of *DNA-dependent protein kinase* (PRKDC), a central node of the cellular DNA damage response system. Activated PRKDC phosphorylates *histone H2AX* (H2AFX) and *cellular tumor antigen p53* (TP53), which subsequently can trigger apoptosis (Cooper et al., 2013). Although the study was controversially discussed (Estaquier et al., 2013), it underscores the far-reaching PTM-based modulations within the cell during viral integration.

Within the context of DNA damage recognition and repair, a study testing the effect of DNA-repair inhibitor caffeine on HIV-1 infection, found evidence for *ATM and Rad3-related kinase* (ATR) being required to successful complete genome integration (Daniel et al., 2003).

The accessory protein VPR is packaged into newly produced virions and plays an important role for early steps in *de novo* viral infection. VPR harbors a series of C-terminal serines (S79, S94, and S96), which have been identified to be important for its nuclear localization and viral replication in macrophages (Agostini et al., 2002). These serines were found to be phosphorylated as assessed by an alanine screen. Phosphorylation of serine residue S79 by *cAMP-dependent protein kinase* (PRKA) affects cell cycle progression (Zhou and Ratner, 2000; Barnitz et al., 2010) and combined mutation of all three serines decreased HIV-1 replication in macrophages (Zhou and Ratner, 2000). Notably, the replication defect was shown to be due to an inefficient nuclear import of viral cDNA as demonstrated by reduced levels of 2-LTR circles (Agostini et al., 2002).

Overall, viral integration is controlled by a multitude of post-transcriptional modifications (**Figure 3**) that modulate function of viral and host cell proteins, thereby affecting enzymatic activity, protein-protein interaction or subcellular localization of integration- related proteins.

### Transcription and Latency

**FIGURE 4:**
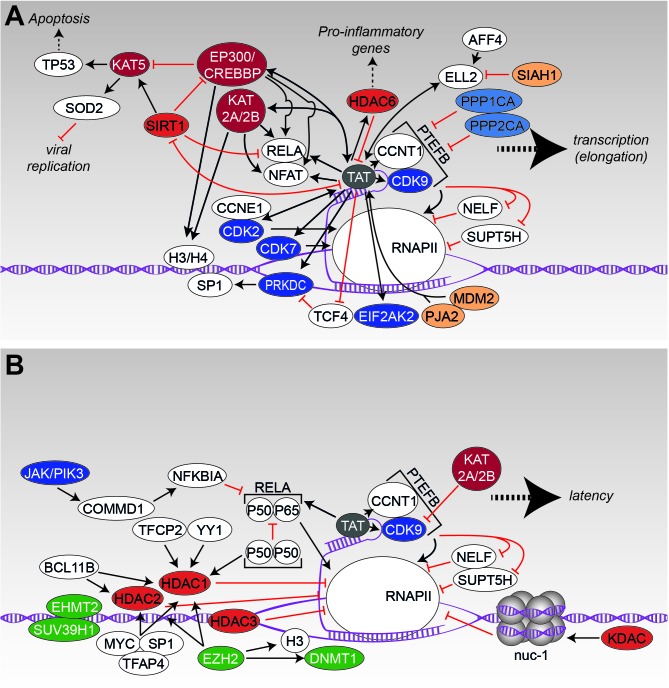
Transcription and latency. **(A)** The interaction scheme displays protein-protein interactions that have been identified to promote transcription of viral genes. **(B)** Likewise, to **A**, the protein network demonstrates the influence of modifying enzymes onto the latent state of HIV. Figure legend as in **Figure 2A**. Additionally, methyl-transferases, depicted in green, are shown. More detailed information can be found in the text.

After integration of the viral cDNA into the host genome, the LTR-flanked provirus behaves like a cellular gene, where the 5^′^-LTR serves as a promoter and the 3^′^-LTR as polyadenylation- and termination-site. Single-molecule amplification and long-read sequencing revealed that transcription from the 5^′^-LTR results in the generation of one primary transcript, which is further spliced into at least 109 different mRNAs (Ocwieja et al., 2012). Depending on the cell-cycle, activation state of the infected cell, integration site, and other factors, HIV either continues to express (**Figure 4A**) or becomes latent (**Figure 4B**). As both processes are interconnected and highly modulated/influenced by post-translationally modified host factors and viral proteins, we will dissect both events to allow a more detailed view on the molecular mechanisms associated with either latency or viral gene expression.

#### Transcription

Immediately after successful integration into the host chromosome, the HIV genome becomes regulated by cellular transcription factors and some viral proteins. The transcription pre-initiation complex, harboring a complex composition of proteins like RNA Polymerase II and some transcription factors, binds to enhancer elements within the 5^′^-LTR and accumulates more essential host transcription factors like RELA (Nabel and Baltimore, 1987), NFAT (Kadonaga et al., 1986), and *transcription factor SP1* (SP1) (Jones et al., 1986) to the viral promoter region. At the beginning, a relatively small number of fully spliced RNA transcripts is generated, which encode for the *regulator of expression of viral proteins* (REV) and the *trans-activator of transcription* (TAT). Subsequently, both proteins are shuttled to the nucleus where they are required for the regulation of viral gene expression and mRNA transport into the cytosol. As long as TAT is absent, transcriptional initiation at the LTR is efficient – however, successful transcription is prevented as the polymerase will either disengage from the DNA template or is stalled in a concerted action of the negative elongation factors (NELFs), *transcription elongation factor SPT5* (SUPT5H) and NELF. Following binding of TAT to the HIV-related RNA stem-loop structure transactivation response element (TAR) (Dingwall et al., 1990), the positive transcription elongation factor b (PTEFB), consisting of the *cyclin-dependent kinase 9* (CDK9) and *cyclin-T1* (CCNT1), is recruited to the RNA polymerase II (RNAPII) complex (Garber et al., 1998; Zhou et al., 2000). Thereby, functional capacity of RNAPII is increased and efficient elongation of transcripts is initiated.

Several of these steps are modulated and fine-tuned by the interplay of PTM modifying enzymes, PTM-interacting proteins, and the viral/host transcription machinery (**Figure 4A**). One of the most studied proteins in this context is the viral TAT protein, which has been shown to interact with more than 180 different nuclear proteins by a proteomic screen (Gautier et al., 2009). Due to this extensive number of complex interactions, we will focus here only on some PTM-related interactions of TAT (a more comprehensive overview of TAT-related interactions is given in **Figures 4A,B**). TAT shapes the nuclear acetylome by recruiting several acetyltransferases (KATs) such as KAT2A/*histone acetyltransferase KAT2B* (KAT2B) and EP300/*CREB-binding protein* (CREBBP) to the 5^′^-LTR to enhance transcription (Lusic et al., 2003; Boehm et al., 2013). Discrete histone regions, on histone 3 (H3) and 4 (H4), were observed to be acetylated using a quantitative chromatin immunoprecipitation assay. Along with histone acetylation was the recruitment of EP300/CREBBP, KAT2B and KAT2A as well as the transcription factor RELA by TAT (Benkirane et al., 1998; Lusic et al., 2003). Moreover, it was found that the P50 subunit as well as the P50/P65 complex of RELA were acetylated by EP300/CREBBP in a TAT-dependent manner. Thereby, acetylation of P50 increased DNA binding of RELA, which coincided with increased transcription rates (Furia et al., 2002). The diverse interactions of TAT are controlled by differential acetylation of TAT itself (Ott et al., 1999; Brès et al., 2002; D’Orso and Frankel, 2009). For example, while acetylation of TAT by EP300 within the TAR binding domain at lysine K50 promotes dissociation of TAT from TAR RNA – an important process in the early steps of transcriptional elongation, acetylation of lysine K28 via KAT2B enhances TAT binding to associated kinases CDK9/PTEFB (Kiernan et al., 1999; Deng et al., 2000; D’Orso and Frankel, 2009). Similarly, KAT2A also considerably enhances the transcriptional activity of TAT by acetylation of lysines K50/K51 (Col et al., 2001). Interestingly, HDAC6 was discovered to deacetylate TAT at lysine K28, thereby suppressing its transactivation ability of the HIV LTR region – a process that on the first view seems to counteract TAT activity but is in fact postulated to be important for transcriptional fine-tuning and RELA activity (Huo et al., 2011). Moreover, it has been reported that TAT increases HDAC6 expression in astrocytes, which results in direct regulation of pro-inflammatory genes (Youn et al., 2015, 2017).

Another complex interaction exists between TAT, the acetyltransferase *histone acetyltransferase KAT5* (KAT5, also known as TIP60), and the *NAD-dependent protein deacetylase sirtuin-1* (SIRT1). TAT causes proteasomal degradation of KAT5 via EP300/CREBBP, preventing TP53-related apoptosis (Harrod et al., 2003; Col et al., 2005; Sapountzi et al., 2006) and additionally disturbing expression of KAT5-dependent genes such as manganese-dependent *superoxide dismutase* (SOD2), an enzyme that normally interferes with efficient virus replication and propagation (Creaven et al., 1999).

However, TAT also inhibits SIRT1, which, in contrast, enforces acetylation of TP53 and thus promotes apoptosis in the host cell (Thakur et al., 2012). Moreover, inhibition of SIRT1 results in hyperacetylation of RELA and EP300/CREBBP, which subsequently leads to activation of RELA-driven transcription (Blazek and Peterlin, 2008; Pinzone et al., 2013) and concomitantly to hyperactivation of the T-cell, a state frequently observed in HIV infected individuals (Kwon et al., 2008). Interestingly in this context, it has also been shown that TAT itself requires deacetylation by SIRT1 for a new binding cycle to the TAR stem-loop, which is necessary for full transcription (Pagans et al., 2005; Blazek and Peterlin, 2008). In summary, findings implicate that the interplay between TAT and SIRT1 requires a fine-tuned spatio-temporal regulation.

Apart from interaction with acetylation-related proteins, TAT interacts with several kinases and phosphatases in order to manipulate cellular pathways. As described before, TAT actively recruits CDK9 and CCNT1 (PTEFB complex) to the TAR RNA loop and induces phosphorylation of serine S5 of the CTD of RNAPII, which enhances co-transcriptional capping of HIV-1 RNA (Herrmann and Rice, 1993; Yang et al., 1996; Zhou et al., 2003). Additionally, CDK9 is recruited to phosphorylate SUPT5H and NELF, thereby alleviating their repressive effect on transcription elongation (Yamaguchi et al., 1999; Rice, 2016). Other CTD kinases like CDK2 and *cyclin-dependent kinase 7* (CDK7) were also reported to be activated by TAT for further phosphorylation of RNAPII (Cujec et al., 1997; Furia et al., 2002). Interestingly, activity of TAT in return seems to be dependent e.g., on phosphorylation of serine residues S16/S46 by the CDK2/*G1/S-specific cyclin-E1* (CCNE1) complex as shown in *in vitro* and in cellula experiments. Mutation of both serines reduced HIV-1 transcription and inhibited viral replication in transiently transfected cells (Deng et al., 2002; Ammosova et al., 2006). Simultaneously, TAT activation of CDK9 is counteracted by *serine/threonine-protein phosphatase PP1-alpha catalytic subunits* PPP1CA and PPP2CA, which cause destabilization of the PTEFB-TAT transactivation complex (Ammosova et al., 2005; Tyagi et al., 2015). TAT has also been shown to induce phosphorylation of SP1 via PRKDC (Chun R.F. et al., 1998). Concurrently, TAT prevents dephosphorylation of SP1 by *transcription factor* *TCF4* (TCF4) in astrocytes as demonstrated by biochemical characterization (Rossi et al., 2006). Another important interaction has been determined between TAT and the *interferon* (IFN*)-induced, double-stranded RNA-activated protein kinase* (EIF2AK2). TAT competes with EIF2AK2’s usual substrate, the *elongation factor 2* (EEF2) and thereby circumvents the IFN-induced translation-block within the host cell. Moreover, interaction with EIF2AK2 results in phosphorylation-dependent enhancement of TAT-based transcription (Endo-Munoz et al., 2005).

As the interplay of TAT with acetylation- and phosphorylation-related proteins for transcriptional fine-tuning and viral amplification has been documented in a variety of experiments, only a small number of interactions with ubiquitin or other PTM modifiers (e.g., SUMO) has been discovered so far. A classical function of ubiquitination is the general protein turnover via proteasomal degradation. Li and colleagues (Zhang et al., 2014) showed that TAT undergoes K48-linked ubiquitination, targeting it to proteasomal degradation. Thereby, TAT’s transactivation activity is modulated. On a more general look, TAT interacts with several E3 ubiquitin-protein ligases. As an example, proto-oncogene MDM2 mediates the ubiquitination of TAT at lysine residue K71 and thus stimulates the transcriptional properties of TAT (Brès et al., 2003). Recently, it has been shown that *E3 ubiquitin-protein ligase Praja-2* (PJA2) can ubiquitinate TAT in a non-degradative manner and specifically regulates HIV transcription elongation (Faust et al., 2017).

Overall, and adding another level of complexity TAT’s multitude interactions are believed to be tightly regulated at different steps of the replication cycle and in individual cell types.

#### Latency

Apart from the active transcription of viral genes described above, also transcriptionally silent and long-lived cell reservoirs are established, which remain the major obstacle to HIV-1 eradication as they stay unaffected by anti-retroviral therapy and are the source of viral rebound upon cessation of antiretroviral therapy (ART) (Chun et al., 1997; Zaikos and Collins, 2014; Lee and Lichterfeld, 2016; Colby et al., 2018). Studies have shown that the reservoir is very stable with a halflife of approximately more than 3 years (Finzi et al., 1999; Siliciano et al., 2003; Crooks et al., 2015). It has been proposed that either a clonal expansion of latently infected cells (Murray et al., 2016) or a persistent low level replication of HIV-1 are responsible for sustainment of the reservoir (Lorenzo-Redondo et al., 2016).

Latency itself is a proportionally rare event in the context of HIV (Siliciano et al., 2003), launching early during acute infection, likely within the range of days (Chun T.-W. et al., 1998). The latent reservoir is estimated to exist with a mean frequency of ∼1/10^6^ resting CD4 T-cells in treated individuals (Eriksson et al., 2013), but could comprise more cells/cell types as the reservoir is incompletely defined (Ho et al., 2013). Latently infected lymphoid cells reside in the host without or very low expression of viral proteins (Folks et al., 1986; Jordan et al., 2003), providing a basis for their immune escape. The reason for the inactive viral state lies mainly in the resting nature of CD4 T-cells. However, despite its clinical relevance, the molecular mechanisms that are responsible for establishment and maintenance of HIV latency remain unresolved. Nevertheless, it is unquestionable that chromatin remodeling of the integrated HIV promoter region has to take place as well as epigenetic silencing and modulation of transcription factor binding to achieve a latent state (**Figure 4B**). Analysis of integration sites in the J-Lat latency cell model system suggests that proviral integration in or close to heterochromatin regions promotes latency (Lewinski et al., 2005). Also integration in the opposite direction to the host gene or intron sites results in antisense transcripts (Landry et al., 2007). In this context, acetylation of histones and histone-associated proteins might play a key role. Usually, acetylation of nuclear proteins directly influences transcription activity by enabling access of the transcription machinery to the promoters. On the contrary, protein-deacetylation apparently restricts transcription factor (TF) binding, which results in gene silencing. This classical rule holds also true for HIV, reflected by a series of studies demonstrating that, besides antigenic stimulation of cytokines, inhibition of KDACs by chemical compounds is sufficient to reactivate HIV and as a consequence to unmask and sensitize a proportion of latently infected cells for killing by ART (Moog et al., 1996; Van Lint et al., 1996; Witvrouw et al., 1997; El Kharroubi et al., 1998; Quivy and Van Lint, 2002; Shirakawa et al., 2013). Further experiments disclosed recruitment of KDACs to the HIV-5^′^-LTR by transcriptional repressors such as the *alpha-globin transcription factor CP2* (TFCP2) or *transcriptional repressor protein YY1* (YY1) (Coull et al., 2000). Interestingly, binding of YY1 to the LTR is directly coupled with the T-cell’s activation state as *T cell receptor* (TCR)-triggered activation causes dissociation of this repressor (Bernhard et al., 2013). Another mechanism of maintaining latency has been discovered for the P50 subunit of RELA. While the P50/P65 heterodimer of RELA is a potent activator of HIV transcription (as discussed earlier), the P50 homodimer serves as an antagonist of transcription. Dimeric P50 recruits HDAC1 to the LTR, promoting chromatin condensation and decreased binding of RNAPII (Williams et al., 2006; Chan and Greene, 2011). The transcriptional repressor *recombination binding protein suppressor of hairless* (RBPJ, also known as CBF-1) is able to redirect HDAC1 to the RELA binding element, promoting maintenance of latency and a lowered association of RNAPII to the LTR region (Tyagi and Karn, 2007; Bernhard et al., 2013). Further (co-)repressors or TFs that recruit HDAC1 and *histone deacetylase 2* (HDAC2) to the LTR and are relevant to establish and endure latency are e.g., *B-cell lymphoma/leukemia 11B* (BCL11B), *transcription factor AP-4* (TFAP4), *Myc proto-oncogene protein* (MYC) or SP1 (Marban et al., 2005, 2007; Imai and Okamoto, 2006; Jiang et al., 2007; Keedy et al., 2009). More evidence for the importance of KDACs for HIV latency was provided by the Margolis laboratory, which demonstrated that (i) HDACs 1, 2, and 3, but not HDACs 4, 5, 7, and 9 associate with the 5^′^-LTR in latency model cell lines, (ii) are expressed in the nuclei of resting CD4 T-cells, (iii) and their chemical inhibition by KDAC inhibitors efficiently induces viral outgrowth (Archin et al., 2009; Keedy et al., 2009). Furthermore, nucleosomes that are located to the LTR region, are able to inhibit the movement of RNAPII (Verdin et al., 1993) – though this block can be overcome by activation of the cell. Here, nuc-1, a nucleosome positioned immediately downstream of the HIV transcription site, has been found to undergo deacetylation by interaction with KDACs in order to maintain the transcriptionally silent state (He and Margolis, 2002; Conrad and Ott, 2016).

Another factor involved in latency control is CDK9. Acetylation of CDK9 by KAT2A/ KAT2B reduces the transcriptional activity of PTEFB, which in turn silences transcription of the provirus (Sabò et al., 2008). Notably, in resting and memory CD4 T-cells, PTEFB levels are already intrinsically low (Rice, 2013), which generally supports their latent state.

In cells of the myeloid lineage, Okada and colleagues identified another mechanism for latency regulation, which is based on phosphorylation instead of lysine-acetylation. Here, the endogenous inhibitor of RELA, *NF-kappa-B inhibitor alpha* (NFKBIA, also known as IκBα), is enhanced by *tyrosine-protein kinase* (JAK)/ *phosphatidylinositol 4,5-bisphosphate 3-kinase* (PIK3)-based induction of the host-derived factor *COMM domain-containing protein 1* (COMMD1), thereby reinforcing latency (Taura et al., 2015). As phosphorylation is well known for its general impact on transcription (Hunter and Karin, 1992) it is not surprising that additional factors are controlled via this PTM in the course of HIV latency. As an example, it was recently shown that a chemical compound, namely the small molecule activator of protein phosphatase 1 (SMAPP1) induced both, HIV-1 latency reactivation and replication. Treatment of cells with SMAPP1 increased phosphorylation of CDK9 and caused upregulation of PTEFB and PPP1CA-related proteins (Tyagi et al., 2015). Moreover, the phosphorylation of histone H1 (H1) via PTEFB is known to be important for the regulation of chromatin binding. Analysis of a phosphorylation-incompetent H1.1 mutant disrupted TAT transactivation and caused reduction of H1 mobility (O’Brien et al., 2010). Low levels of PTEFB promote the non-mobile condition of H1. Another latency-promoting factor regulated by phosphorylation is the *signal transducer and activator of transcription 5* (STAT5). Compound screens revealed that inhibition of a negative feedback loop, that usually sumoylates phosphorylated STAT5, sustained its phosphorylated state. This increases activity and occupancy of the HIV-1 LTR and subsequently causes latency reversal (Bosque et al., 2017).

Other PTMs directly related to HIV latency are protein-methylation and -ubiquitination, which will be discussed only briefly in this review. Trimethylated histones are a well-known modification in repressive heterochromatin (Stewart et al., 2005; Saksouk et al., 2015) and, as expected, have been observed for 5^′^-LTR associated nucleosomes during latency (du Chéné et al., 2007; Marban et al., 2007; Pearson et al., 2008). Consequently, several accompanied histone methyltransferases (HMTs) have been identified to reside at latent proviral 5^′^-LTRs. Examples are the *histone-lysine N-methyltransferases SUV39H* (SUV39H1), *EHMT2* (EHMT2) or the *EZH2* (EZH2) (du Chéné et al., 2007; Marban et al., 2007; Imai et al., 2010; Friedman et al., 2011; Mbonye and Karn, 2014). Especially EZH2 has been proposed as one of the major regulators of latency, as it not only methylates H3 on lysine K27, but also resembles the catalytic subunit of polycomb repressive complex 2 (PRC2). This complex (together with PRC1) localizes at specific sites called polycomb repressive elements and organizes the chromatin into a repressive structure that is unresponsive to chromatin remodeling factors and basal- and gene-specific transcription factors (Otte and Kwaks, 2003). Moreover, PRC2 has been shown to recruit several repressive chromatin modifiers like *DNA (cytosine-5)-methyltransferase 1* (DNMT1) as well as HDAC1 and 2 (Van Der Vlag and Otte, 1999; Tonini et al., 2004; Montgomery et al., 2005).

An example for the involvement of ubiquitination in latency, is the *E3 ubiquitin-protein ligase SIAH1* (SIAH1), which has been identified by Zhou and colleagues (Liu et al., 2012) as the E3 ubiquitin ligase for *elongation factor for RNA polymerase II 2* (ELL2). ELL2 forms together with ELL1, TAT, CCNT1, CDK9, *AF4/FMR2 Family Members 1 and 4* (AFF1/4) and other members the super elongation complex (SEC), which is required for active transcription. SIAH1 causes polyubiquitination and subsequent proteasomal degradation of ELL2 in actively replicating cells and absence of ELL2 strongly inhibits TAT-related gene expression (He et al., 2010). Interestingly, both, activated TAT and the SEC scaffold protein AFF4, prevent degradation of ELL2 leading to a more profound transcription of viral genes (Liu et al., 2012). Taken together, the interplay of several factors – mainly located close to or recruited to the 5^′^-LTR – is able to promote or prevent latency (**Figures 4A,B**). Thereby, not only the integration site, but also local protein concentrations together with different PTMs control a positive or negative transcriptional environment for the provirus.

### Translation, Assembly, Budding and Final Maturation

**FIGURE 5:**
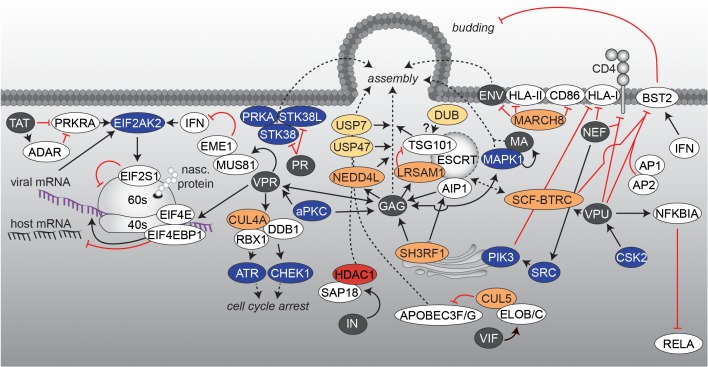
Viral protein translation and virus assembly. The interaction network visualizes processes, which are accompanied with the late steps of HIV infection and the formation of new virions. Figure legend as in **Figure 2A**. More detailed information can be found in the text.

At present, insights into the involvement of PTMs during the last steps of the HIV replication cycle are limited. Viruses completely rely on the host protein synthesis machinery – consisting of ribosomes, tRNAs, amino acids and associated factors for translation-initiation, -elongation and -termination. Moreover, innate antiviral restriction factors as well as stress responses have to be circumvented and at the same time the cellular tRNA pool has to be used in an efficient way. Additionally, intracellular transport networks and cofactors have to be exploited for virion assembly and particle maturation, respectively. All these requirements necessitate an interplay of the virus with specific cellular pathways, which are undoubtedly regulated via PTMs (**Figure 5**). One of these counteractions is based on the production of IFNs upon host’s detection of viral encounters. Expression of IFN in combination with the presence of viral RNAs leads to induction and activation of EIF2AK2, which phosphorylates several substrates including *eukaryotic translation initiation factor 2 subunit 1* (EIF2S1). This modification shuts down general synthesis of proteins in order to prevent viral protein production. Here, HIV-1 TAT compromises the function of *IFN-inducible double-stranded RNA-dependent protein kinase activator A* (PRKRA) to block EIF2AK2 activation and thus, the shut-down of protein translation (Clerzius et al., 2013; Burugu et al., 2014; Chukwurah et al., 2017). At the same time, the viral factor VPR takes over the host CUL4/DDB1/*E3 ubiquitin-protein ligase* *RBX1* (RBX1) complex, finally leading to activation of the *Serine/threonine-protein kinases ATR* (ATR) and *Chk1* (CHEK1) for prevention of cell cycle progression into mitosis and induction of G2/M arrest in HIV infected cells. Furthermore, VPR activates the host *crossover junction endonucleases MUS81* (MUS81) and *EME1* (EME1) via polyubiquitination of MUS81, which blocks interferon stimulated gene (ISG) expression(Roshal et al., 2003; Laguette et al., 2014; Zhou et al., 2016). Several lines of evidence also demonstrated a selective suppression of mRNA translation in lymphocytes accompanied with VPR-induced cell cycle arrest. The molecular basis for this effect seems to be related to reduced levels of the phosphorylated *eukaryotic translation initiation factor 4E* (EIF4E) and *EIF4E-binding protein 1* (EIF4EBP1) (Sharma et al., 2012) (**Figure 5**). Interestingly, due to composition of the viral mRNA-ribonucleoprotein complexes, harboring components of the nuclear cap binding complex (CBC), viral protein synthesis is sustained in comparison to suppressed host protein translation (Sharma et al., 2012). After translation of viral proteins, exploitation of the host protein sorting and trafficking pathways becomes crucial for assembly of virions. For this, as in the early steps of infection, the actin-tubulin-network has to be altered (Jolly et al., 2007), which is likely accompanied with acetylation, phosphorylation, and ubiquitination events. Indeed, ubiquitination is involved in the sorting of HIV proteins. For example, the human *trans*-Golgi network (TGN) associated *E3 ubiquitin-protein ligase* *SH3RF1* (SH3RF1) was identified as an important sorting factor for GAG’s localization to the plasma membrane (Alroy et al., 2005). Further experiments identified *programmed cell death 6-interacting protein* (PDCD6IP, also known as AIP1), a member of the endosomal sorting complex required for transport (ESCRT) machinery, as substrate of SH3RF1, which is obligatory for HIV-1 release (Votteler et al., 2009). Another E3 ubiquitin ligase that is involved in the regulation of GAG transport to the cell surface is the *susceptibility gene 101 protein* *TSG101* (TSG101) associated *E3 ubiquitin-protein ligase LRSAM1* (LRSAM1) (Amit et al., 2004). TSG101 belongs to the ESCRT complex and is engaged by GAG for efficient HIV-1 budding (**Figure 5**). LRSAM1-mediated ubiquitination of TSG101 inactivates its sorting function. Presumably, by the coordinated action of a DUB, TSG101 gets recycled and a second round of cargo-loading is enabled (Amit et al., 2004). Similarly, GAG interacts with the *E3 ubiquitin-protein ligase NEDD4-like* (NEDD4L), which stimulates in liaison with TSG101 the release of HIV virions (Chung et al., 2008). Due to technical limitations, the identification of specific DUBs relevant for HIV remains unknown. However, recently, Setz et al. could show that *ubiquitin carboxyl-terminal hydrolases 7* (USP7)- and *47* (USP47)-specific inhibitors P22077 and PR-619 are able to impair GAG processing and affect infectivity of released HIV virions, underscoring the importance of DUBs in HIV biology (Setz et al., 2017). Expectedly, cellular countermeasures have evolved utilizing the ubiquitination system to inhibit virus assembly. One example is the *E3 ubiquitin-protein ligase MARCH8* (MARCH8), which downregulates several host transmembrane proteins including *HLA class II histocompatibility antigen (*HLA-II, also known as MHC class II*)* or *T-lymphocyte activation antigen CD86* (CD86). Experiments identified MARCH8 as a new restriction factor that blocks the incorporation of HIV-1 envelope glycoprotein into virus particles by downregulating it from the cell surface (Tada et al., 2015). Other PTMs are also involved in cellular localization of GAG molecules and correct assembly of HIV particles. Using a proteomic approach, phosphorylation of GAG by the PRKC was detected. This phosphorylation event is necessary for the interaction between GAG and VPR and results in the incorporation of VPR into the virions (Kudoh et al., 2014). A second kinase that regulates viral assembly and release via GAG phosphorylation is MAPK1 (Hemonnot et al., 2004). MAPK1 phosphorylates the MA/P17 of HIV and gets itself incorporated into newly formed virions (Jacqué et al., 1998). However, the precise mode of action remains to be explored. Several other serine-threonine kinases are directly incorporated into the virions as well, such as PRKA (Cartier et al., 2003) and *serine-threonine kinase 38*/*38 Like* (STK38/STK38L), respectively (Devroe et al., 2005). STK38 and STK38L were found to be proteolytically processed by the viral protease PR in the virions as well as within infected producer cells. Truncation at the PR cleavage site altered subcellular localization of STK38L and inhibited the enzymatic activity of STK38 and STK38L (Devroe et al., 2005). In addition, viral integrase IN binds SAP18 and supports incorporation into progeny viral particles together with other compounds of the SIN3A/HDAC1 complex (**Figure 5**). Fluorescence-based assays revealed significant KDAC activity of virions and demonstrated a positive effect of HDAC1 for infectivity (Sorin et al., 2009).

Besides facilitating virion assembly and co-incorporation, viral proteins are also active in depletion or inactivation of antiviral factors, which counteract particle infectivity. During particle generation, the viral protein VIF is found in the cytoplasm to employ different strategies for antagonizing APOBEC3F/3G (**Figure 5**). In the absence of VIF, APOBEC3G or APOBEC3F become incorporated into budding virions and impede reverse transcription and integration in a newly infected cell (see also uncoating & RT) (Harris et al., 2003; Mangeat et al., 2003; Zhang et al., 2003; Bishop et al., 2004; Liddament et al., 2004; Yu et al., 2004). VIF inhibits APOBEC3G packaging by competing with binding to viral RNA or the nucleocapsid (Kao et al., 2003; Mariani et al., 2003). Furthermore, VIF recruits the cellular E3 ligase *cullin-5* (CUL5), *elongin B* and *C* (ELOB/ELOC), and RBX1 to form a SCF-like complex for proteasomal degradation of APOBEC3G, a process that is further controlled by phosphorylation events within the complex (Conticello et al., 2003; Marin et al., 2003; Stopak et al., 2003; Yu, 2003; Mehle et al., 2004).

Another factor, playing an important role in viral restriction and being antagonized by a viral factor during assembly is the *bone marrow stromal antigen 2* (BST2, also known as tetherin). The interferon-induced protein BST2 impedes detachment of assembling viral particles at the cell surface of the producer cell (Neil et al., 2008; Van Damme et al., 2008). The viral membrane phospho-protein VPU [in some cases also the *negative regulatory factor* (NEF) or ENV (HIV-2)] hijacks the cellular BST2 trafficking and turnover machinery, thereby facilitating viral release (Neil et al., 2008; Van Damme et al., 2008; Sauter et al., 2009; Hauser et al., 2010; Kluge et al., 2014; Arias et al., 2016) (**Figure 5**). Under steady-state conditions, BST2 cycles between the plasma membrane, the *trans*-Golgi Network and endosomes, with a fraction getting sorted to lysosomal degradation via the ESCRT pathway. Studies demonstrated that, mediated by CSK2 phosphorylation (Schubert et al., 1992, 1994), VPU complexes with BST2 and binds to the *clathrin-associated adaptor protein complexes 1* (AP1) or *2* (AP2) (Kueck et al., 2015), which restrains BST2 from its mode of action. Moreover, phosphorylated VPU recruits the SCF-*F-box/WD repeat-containing protein 1A* (BTRC) E3 ligase complex, which leads to ubiquitination of BST2 and thus an enhanced recruitment of BST2 by the ESCRT machinery and subsequent degradation (Douglas et al., 2009; Mangeat et al., 2009; Mitchell et al., 2009; Janvier et al., 2011; Tokarev et al., 2011; Agromayor et al., 2012). Another function of VPU is to block RELA signaling in the late state of the HIV replication cycle, as RELA also regulates the antimicrobial immune response, including the expression of ISGs that protect against viral pathogens (Pfeffer, 2011). It has been shown that while NEF increases RELA activity (as described earlier), VPU stabilizes NFKBIA by preventing its polyubiquitination and degradation via sequestration of the adaptor protein BTRC (Kroll et al., 1999; Akari et al., 2001; Bour et al., 2001; Sauter et al., 2015). Thereby, nuclear translocation of P65 and subsequent RELA activation is impeded (Sauter et al., 2015). To note, it has been reported that BST2 displays an additional innate sensing function, which activates RELA-driven antiviral immune responses and thus being also affected by VPU’s anti-BST2 activity (Cocka and Bates, 2012; Galão et al., 2012; Tokarev et al., 2013).

A further example is the exploitation of the host cell membrane-proteome to avoid detection by the immune system. As production of viral proteins is accompanied by defective ribosomal products (DRiPs) (Yewdell et al., 2001), which can end up as antigenic peptides presented on HLA class I (HLA-I) molecules to report cell infection, viruses have adapted specific mechanisms to circumvent cytotoxic T-cell recognition. In case of HIV, NEF interferes with the normal trafficking pathway of HLA-I and thus reduces recognition and lysis of infected cells by cytotoxic T cells (Wonderlich et al., 2011) (**Figure 5**). Currently, two models are discussed, which might take place at different times of the replication cycle. In the first model, NEF binds and activates TGN-specific SRC-family kinases, which subsequently trigger PIK3-dependent endocytosis of cell surface HLA-I (Blagoveshchenskaya et al., 2002). In the second model, NEF directly interacts with the cytoplasmic tail of HLA-I for shuttling to the lysosomal compartment (Schaefer et al., 2008). In addition, NEF as well as VPU, target the surface-expressed CD4 molecules for downregulation (Guy et al., 1987; Willey et al., 1992). Thereby, NEF induces endocytosis by linking the cytosolic tail of CD4 to components of the cellular protein trafficking machinery (Chaudhuri et al., 2007). In contrast, VPU targets newly synthesized CD4 for ubiquitination in an Endoplasmic Reticulum associated protein degradation (ERAD)-related pathway at the ER level, which also involves recruitment of the SCF-BTRC E3 ligase complex (Willey et al., 1992; Margottin et al., 1998; Magadán et al., 2010). Downregulation of surface CD4 lowers the risk of superinfection of the cell and promotes viral egress as it prevents retention of newly assembled virions at the cell surface (Ross et al., 1999). All these observations demonstrate the importance of PTMs within the final steps of HIV assembly and release (see also **Figure 5**). However, a more comprehensive picture of the late molecular steps of the replication cycle requires a further focus on PTM-related processes.

## Conclusion and Future Outlook

Given the broad role of PTMs in every step of the HIV replication cycle and by controlling and fine-tuning almost every cellular process, it is likely that many additional interactions will be discovered in future. Recent methodological advances in the field of mass spectrometry-based proteome-wide screens will allow for the first time broad-range and unbiased investigations of changes in the global PTM landscape. Moreover, new and sensitive methods for label-free quantitative interaction-proteomics in primary cells have become available, which permit detection of weak interactors with spatial and temporal resolution. These developments in combination with thorough follow-up studies will hopefully help to identify vulnerable nodes of virus-host interaction, facilitating so far unappreciated therapeutic approaches for the treatment of HIV infected individuals or protection of those at risk of infection. Moreover, many discoveries might be transferable to other scientific fields enabling the generation of new molecular tools for basic research as well as for the treatment of other epidemics. We hope that this review helps to arouse the interest in PTM-related virological research and apologize to all investigators in the field for the arbitrary selection of processes, covering only a small part of important scientific findings.

## Author Contributions

LC, OK, and CS wrote the manuscript. LC and CS compiled data sets and created the figures.

## Conflict of Interest Statement

The authors declare that the research was conducted in the absence of any commercial or financial relationships that could be construed as a potential conflict of interest.

## Footnotes

1 www.genenames.org

2 www.kinase.com

3 www.depod.bioss.uni-freiburg.de
